# Phyllodes tumour of the breast

**DOI:** 10.2349/biij.2.2.e33

**Published:** 2006-04-01

**Authors:** M Muttarak, P Lerttumnongtum, A Somwangjaroen, B Chaiwun

**Affiliations:** 1 Department of Radiology, Chiang Mai University, Chiang Mai, Thailand; 2 Department of Surgery, Chiang Mai University, Chiang Mai, Thailand; 3 Department of Pathology, Chiang Mai University, Chiang Mai, Thailand

## HISTORY

A 46-year-old woman presented with a painless palpable mass in the left breast for two weeks. She had no nipple discharge and no familial history of breast carcinoma. Physical examination revealed a 4.5-cm circumscribed, movable mass in the left upper outer quadrant. The overlying skin was normal. The axillary lymph node was not enlarged.

## IMAGING FINDINGS

**Figure 1:**
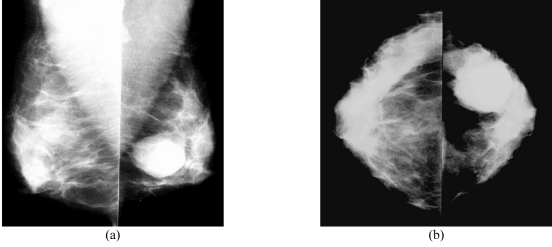
(A) Mediolateral oblique and (B) craniocaudal mammograms show a heterogeneously-dense breast with a round, well-circumscribed, 4.5-cm mass at 3 o’clock in the left breast.

**Figure 2:**
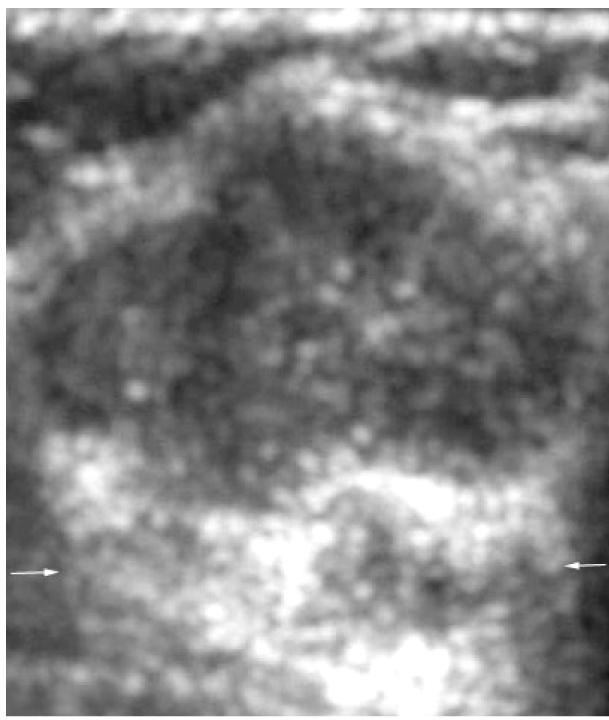
Transverse US image shows a circumscribed, lobulated mass with heterogeneous internal echoes and a slight posterior acoustic enhancement (arrows).

Mammograms revealed a 4.5 cm, well-circumscribed mass without calcification at 3 o’clock in the left breast (Figure 1). Ultrasonography (US) revealed a circumscribed, macro lobulated mass with heterogenous internal echoes and a slight posterior acoustic enhancement (Figure 2).

## CLINICAL COURSE

Fine-needle aspiration biopsy (FNAB) of the mass showed benign epithelial cells which could be either fibroadenoma or phyllodes tumour. The patient underwent a wide excision of the mass. She made an uneventful recovery, and a simple mastectomy was planned.

## PATHOLOGICAL FINDINGS

**Figure 3:**
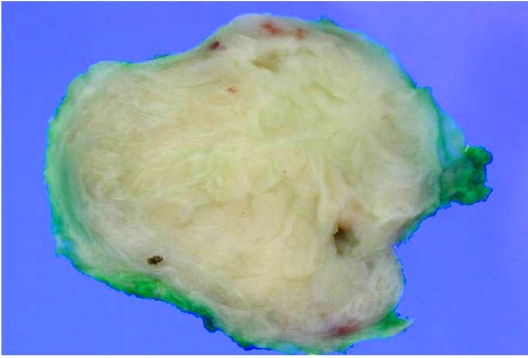
Photograph of an excised specimen shows a well-circumscribed, macrolobulated mass with greyish-white trabeculated cut surface.

**Figure 4:**
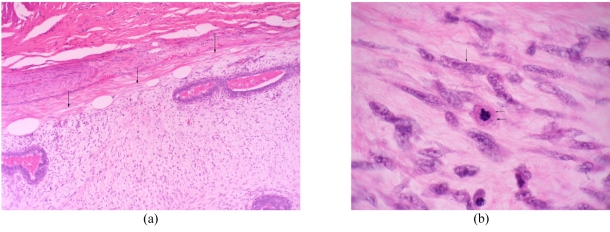
(A) Photomicrograph shows circumscribed border of tumour (arrows) (Haematoxylin & eosin stain, X40). (B) Photomicrograph shows spindle cells with plump nuclei (arrow). Mitosis (double arrows) is also noted. (Haematoxylin & eosin stain, X400).

At gross examination, the specimen contained a circumscribed mass measuring 4.5 cm in diameter with grayish-white trabeculated cut surface (Figure 3). Microscopic examination revealed long attenuated ducts among cellular stroma with circumscribed border (Figure 4a). The stroma consisted of spindle-shaped cells with elongated plump nuclei. Some nuclei were pleomorphic (Figure 4b). Mitotic figures were occasionally observed, approximately more than 5 mitoses per high-powered field on average. These findings were consistent with malignant phyllodes tumour.

## DISCUSSION

**Figure 5:**
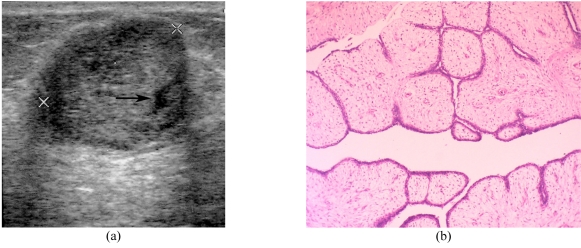
Benign phyllodes tumour in a 35-year-old woman. (a) Transverse US image shows a circumscribed heterogenous echo with a small cystic space (arrow) and a slight posterior acoustic enhancement. (b) Photomicrograph shows leaf-like processes containing cellular stroma lined with benign ductal epithelial cells projecting into the cystic space (haematoxylin & eosin stain; x100).

**Figure 6:**
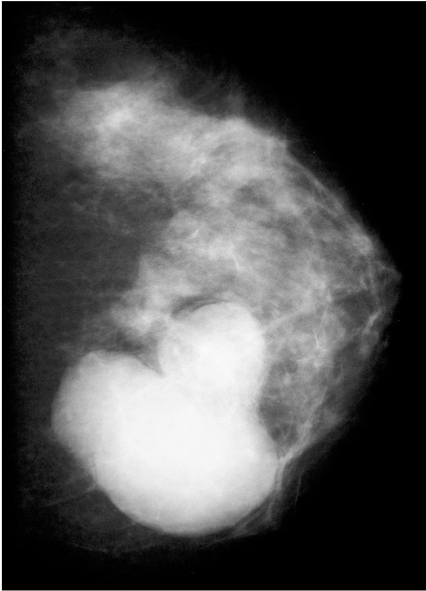
Benign phyllodes tumour in a 48-year-old woman. Left craniocaudal mammogram shows a 6-cm lobulated, circumscribed mass in the inner quadrant.

Phyllodes tumour, previously described by Johannes Muller in 1838 as cystosarcoma phyllodes [1], accounts for less than 1% of mammary tumours and represents approximately 2%-3% of fibroepithelial tumours of the breast [2]. Phyllodes tumour is composed of epithelial elements and a connective tissue similar to fibroadenoma but phyllodes tumour has higher stromal cellularity. The tumour usually occurs among women 40-50 years old [3] whereas fibroadenoma is common in women 20-30 years old [2]. Clinically, patients present with a palpable, painless, slow growing mass, that can reach a large size suddenly [4-6]. Occasionally, ulceration of the skin may occur due to stretching over the large tumour. On mammography, phyllodes tumour is seen as a lobulated, round, or oval circumscribed mass without calcification. On US, phyllodes tumour usually appears as a well-defined mass with heterogenous internal echoes and sometimes having posterior acoustic enhancement [4-8]. The presence of fluid-filled, elongated spaces or clefts (Figure 5) within a solid mass is suggestive of phyllodes tumour but not pathognomonic of the diagnosis [5,6]. Liberman et al [7] reported that a phyllodes tumour with diameter greater than 3 cm tended to be associated with malignancy. However, there are no reliable mammographic or US features to differentiate benign from malignant phyllodes tumour [4-6,8] (Figure 6). Differentiation of phyllodes tumour from fibroadenoma by mammographic and US features is difficult but important because of difference in management [3,5]. Fibroadenoma may regress spontaneously so follow-up in selected women such as those who are young without high risk of breast cancer, pregnant or refuse surgery is possible [3]. Whereas, phyllodes tumour requires complete surgical removal of the mass with wide margins.

Preoperative diagnosis of phyllodes tumour with FNAB is controversial because fibroadenoma is frequently diagnosed due to the substantial cytologic overlap similar to our presented. Occasionally, false-positive diagnosis of carcinoma is also made [4,5]. Multiple samplings are required for a correct diagnosis because phyllodes tumour is often heterogeneous. Since it is difficult to differentiate fibroadenoma from phyllodes tumour on imaging features and cytology, histological examination should be conducted to confirm the diagnosis. The distinction between them bases solely on the histologic features of stroma [5]. Phyllodes tumour may be classified as benign, borderline or malignant [6,7]. Although phyllodes tumour is usually benign, approximately 20-50% are malignant. Histological indications of malignancy include increased mitotic activity, pronounced proliferation of stromal components relative to glandular structures, cytologic atypia, and invasive peripheral growth with infiltration into adjacent tissues [6]. Distant metastases occur less than 20%, mainly in malignant phyllodes tumour but have also been reported in benign ones [4,6,7]. Metastatic tumour spread is primarily haematogenous, most commonly to lung, pleura and bone. Fewer than 1% of malignant phyllodes tumour spread to axillary lymph node [9].

Treatment of phyllodes tumour requires complete removal of the tumour with wide margins if the tumour is small and a simple mastectomy may require if the tumour is large. Local recurrence occurs in approximately 20% of cases if the tumour is incompletely excised [3,7]. Routine axillary node dissection does not appear to be indicated [10]. A combination of surgery, radiation therapy, chemotherapy, and even hormonal therapy is controversial for malignant phyllodes tumour [5].
